# Impact of Ecological Momentary Assessment Participation on Short-Term Smoking Cessation: quitSTART Ecological Momentary Assessment Incentivization Randomized Trial

**DOI:** 10.2196/67630

**Published:** 2025-07-18

**Authors:** Kara P Wiseman, Alex Budenz, Leeann Siegel, Yvonne M Prutzman

**Affiliations:** 1 Department of Public Health Sciences, School of Medicine, University of Virginia, P.O. Box 800765, Charlottesville, VA, 22908, United States, 1 4342438126; 2 Tobacco Control Research Branch, Behavioral Research Program, Division of Cancer Control and Population Sciences, National Cancer Institute, Bethesda, MD, United States

**Keywords:** mHealth, smoking cessation, ecological momentary assessment, EMA, smoking, smoker, quit, cigarettes, vape, e-cigarette, randomized controlled trial, cessation intervention, app, smartphone, mobile phone

## Abstract

**Background:**

Cigarette smoking is the leading cause of preventable mortality in the United States. Cessation interventions delivered through smartphone apps can reach large populations of individuals who smoke. Ecological momentary assessment (EMA), a feature often included in existing cessation apps, can be used to track behaviors and other important constructs and to inform just-in-time interventions. However, the isolated influence of EMA engagement on smoking cessation is unknown. In addition, the implications of incentivizing the use of EMA for cessation outcomes are currently unknown. The National Cancer Institute’s publicly available smoking cessation app, quitSTART, includes a 2-week voluntary EMA protocol (42 total EMA prompts), which provides an opportunity to explore the impact of EMA incentivization on smoking cessation.

**Objective:**

This study aimed to examine the influence of app-based EMA participation on smoking cessation for people who are incentivized to use EMA compared with those who are not incentivized (representing the current implementation of EMA within quitSTART).

**Methods:**

In total, 152 US adults were recruited from web, social media, and SMS text message sources into a randomized controlled trial. All eligible participants were randomized to either nonincentivized EMA or incentivized EMA. Participants completed baseline, 2-week, and 4-week assessments. The primary outcome of interest was 7-day point prevalence abstinence measured at 2 and 4 weeks after app download. Average EMAs completed by arm were compared using a *t* test. Firth logistic regression modeling was used to determine the association between arm and smoking abstinence at 2 and 4 weeks, adjusted for smoking frequency and concurrent use of other tobacco products.

**Results:**

The mean number of EMAs completed was 13.3 (range 0‐40, SD 11.2) in the incentivized arm and 4.7 (range 0‐28, SD 5.8) in the nonincentivized arm (*P*<.001). Cessation rates were 9% and 20.3% at 2 weeks (*P*=.06), and 17.5% and 36.6% at 4 weeks (*P*=.01) in the incentivized arm and nonincentivized arm, respectively. Study arm was not associated with cessation in the adjusted models (adjusted odds ratio [OR] at 2 weeks 0.60, 95% CI 0.21‐1.73; adjusted OR at 4 weeks 0.51, 95% CI 0.22‐1.19).

**Conclusions:**

This study attempted to isolate and examine the effect of incentivizing EMA engagement on smoking cessation success for adults using a smartphone app to quit. While participants randomized to incentivization of EMA showed higher engagement with this feature, our findings suggest that there was no additional short-term cessation benefit from this engagement. Crude analyses found a potential benefit for allowing autonomy over the use of app features, despite the ability of EMA completion to provide real-time, tailored cessation support.

## Introduction

### Background

Cigarette smoking is the leading cause of preventable mortality in the United States and causes multiple cancers, including lung, colorectal, and liver cancers [1-3]. Approximately 28.3 million US adults currently smoke cigarettes [3]. Although more than 50% of people who smoke attempt to quit smoking each year, only 8% are successful [4]. The use of evidence-based cessation treatment (eg, cessation medications and cessation counseling) can substantially improve the odds of successfully quitting [5]. However, most people try to quit smoking without assistance [6-8], which has been shown to be less effective than quitting with assistance [9]. Therefore, connecting people who want to quit smoking with smoking cessation treatment is an important public health goal [8].

Smoking cessation programs using mobile health (mHealth) technologies have the potential to cost-effectively reach individuals who smoke at a population level [10-12]. Specifically, mHealth cessation interventions delivered through smartphones can reach a large population of individuals who smoke in the United States, as 90% of US adults own a smartphone [13]. Smartphone use is also prevalent across sociodemographic groups in the United States [13], which can help to deliver interventions to a diverse population and reduce smoking-related health disparities [14]. The most recent examination of smoking cessation apps found over 500 English language apps are available to smartphone users, and at the time of the study, these apps had approximately 3.2 million US-based downloads [15].

Several studies have demonstrated the effectiveness of smartphone apps in aiding smoking cessation [15-20]. Yet, little is known about the influence of use of specific app features (eg, self-monitoring of smoking and mood, games, and mindfulness training) on smoking cessation. Limited evidence suggests that engaging with certain features, like tracking cravings and engagement with quit plans, can positively influence cessation outcomes [21,22]. In addition, tracking (eg, of smoking behaviors, cravings, and quit progress) is the most frequently reported feature included in smoking cessation apps [23]. Therefore, it is imperative that we understand the effect of engagement with tracking features on cessation outcomes.

One such app feature that can provide tracking and insight into areas such as smoking behavior, key contextual factors associated with smoking, and environmental influences on smoking behaviors is app-based ecological momentary assessments (EMAs) [24]. EMAs involve frequent, repeated sampling of individuals’ environments, affective states, and behaviors [18]. One advantage of using EMAs is that they provide data from participants in their natural environments, giving access to responses that may not be accurately collected in a research setting (eg, using periodic mailed questionnaires). EMAs can also reduce biases related to retrospective recall by collecting real-time data [24,25]. EMA can be used to support self-monitoring, which is hypothesized to support behavior change by providing information about the target behavior (eg, diet, physical activity, and smoking cessation) to the user, fostering reflection and accountability, and raising awareness and consciousness around the target behavior [26]. Therefore, increased use of self-monitoring via EMA is hypothesized to increase the target behavior. Increasingly, EMAs are being leveraged to inform the development of just-in-time adaptive interventions (JITAI) [27], which involve the provision of adaptive support to individuals at a particular time of day, location, or moment of intervention need, using data collected through EMAs or sensors (eg, exercise tracking watches and heart rate monitors) [28]. In the absence of sensors, self-reported data captured through responses to EMAs is the primary source of information available to inform JITAIs, making high engagement with EMAs imperative to support the provision of just-in-time content.

To date, many smartphone-based cessation interventions have incorporated EMA functionality in some capacity within a smoking cessation app [16,18,19,29-37]. Collectively, these studies show promise for the potential of apps that integrate EMA as a cessation strategy. However, questions remain about how EMA engagement specifically impacts cessation outcomes. Importantly, in many cases where EMA has been used as part of the intervention design, both study arms have been asked to participate in the EMA protocol. For example, in a study conducted by Garrison et al [32], participants in both the intervention and control group received EMA prompts 6 times a day for 22 days asking all participants to report current activities in progress, awareness, focus, feelings, cigarette cravings, mood, and number of cigarettes smoked. Schwaninger et al [33] encouraged all participants to complete a daily EMA of exhaled carbon monoxide. Bricker et al [38] compared a cessation app that included EMA (iCanQuit) to the National Cancer Institute QuitGuide app, which does not include EMA, potentially providing some evidence about the impact of EMA on cessation. However, there were multiple differences between the 2 apps, making it difficult to isolate the features that made iCanQuit more efficacious than QuitGuide [38]. To our knowledge, no study has directly compared cessation outcomes within a single app by EMA participation levels. Therefore, the isolated effect of EMA engagement on smoking cessation is unknown.

Adding to these gaps in research is a lack of information about real-world smartphone-based EMA participation and smoking cessation. When implemented in a real-world setting, mHealth app use in general is low [39], but EMA engagement specifically has not been considered. Knowing the importance of EMA engagement on cessation outcomes can help inform whether EMA participation should be incentivized when current and future smartphone cessation apps are disseminated to the public. Consideration about how or to what extent EMA participation has been encouraged in previous studies can help estimate the potential resources necessary if there is a need for continued incentivization of EMA engagement when apps with EMA are made available to the public. Within the existing literature examining the efficacy of smoking cessation apps, several studies have specifically described strategies used to encourage and maintain engagement in EMA completion [15,16,28,30,35,39]. For example, in a study by Businelle et al [18], all participants were incentivized to complete the EMA protocol by having part of their participation incentive linked to EMA completion levels, with a baseline threshold of 50% completed to receive any EMA payment [18,29]. For Garrison et al [32], if participants decreased their EMA participation in the EMA protocol to a prespecified level, they would receive communication from study staff to encourage participation in EMAs. For the iCanQuit study, staff were not involved in encouraging EMA participation, but in order for participants to gain access to specific content, continued smoking abstinence had to be reported through daily tracking, thereby encouraging use of tracking within the app [17,38,40]. Therefore, the data that have been generated from these clinical trials have limited utility for understanding app-based EMA participation when it is not incentivized or the effect of incentivizing the use of EMA on cessation outcomes.

### quitSTART Cessation App

The National Cancer Institute’s Smokefree.gov Initiative provides free, publicly available smoking cessation resources to help people successfully quit [41]. One of these resources is quitSTART, a publicly available smoking cessation app [42]. Available to download on iOS [Apple Inc] and Android [Google] since 2013, the app is widely used, with 1000‐2000 people downloading quitSTART each month. quitSTART provides intervention content in several ways. The user has access to sets of content, called “cards,” that are grouped into categories of information about smoking and health, cessation tips, inspirational quotes, and challenges; cards can be added into a user’s “quit kit” to create a quit plan. Users can proactively (eg, without responding to a prompt or push notification) create app entries to track cravings to smoke, if they smoked, and if they are “feeling down” or “feeling good” [42]. Users who report a craving or smoking can “tag” these actions to a specific time of day or their current location and then receive supportive messages via push notifications during that time of day or the next time they return to a tagged location. quitSTART has not been tested in an efficacy study but has been deemed to be high-quality based on clinical practice guidelines and acceptable in several populations [36,43-46]. A previous randomized pilot study of quitSTART among adolescents who smoked reported 7-day abstinence rates of 16.3% at 6 months [36].

Starting in October 2017, built-in EMA capability was added to quitSTART. The EMA protocol is as follows: starting on the date of download, quitSTART users are automatically opted in to the EMA protocol but may opt out by turning off push notifications. During the first 2 weeks after app download, users are sent 3 EMA completion prompts a day for 2 weeks (n=42 total prompts sent). Users receive prompts at random times during 3 time periods each day and have 1 hour to respond after a prompt is sent. Prompts assess craving level, mood, and number of cigarettes smoked. Users who respond receive a tailored message based on their mood and craving level (Multimedia Appendix 1). Previous descriptive evaluations of voluntary use of EMA within quitSTART have shown very low initial uptake. Specifically, between January and March 2018, 59% (669/1143) of quitSTART users had never completed an EMA prompt. Of those who completed any EMAs, 42% (474/1143) only completed 1 EMA prompt [47]. It is unknown whether the levels of EMA participation in this publicly available smoking cessation app impact smoking cessation outcomes. Therefore, the goal of this study was to isolate the effect of incentivization of app-based EMA engagement on levels of EMA participation and smoking cessation using a randomized controlled trial. Given the reported cessation outcomes of existing apps that use EMA, we hypothesized that those randomized to receive incentives for completing EMAs would have increased EMA engagement and higher odds of self-reported smoking cessation rates at 2 and 4 weeks after app download.

## Methods

### Design

This randomized trial included 152 adults aged 18 years and older who smoked cigarettes. The recruitment period took place between October 2020 and April 2021. Study information presented to all potentially interested participants specified that an incentive for participation would be provided, along with the total potential dollar amount, but without providing any further details. Eligible participants were randomized before consenting and were then sent a consent form specific to their condition. Specifically, participants were randomized to 1 of the 2 conditions: nonincentivized EMA (control), or incentivized EMA (refer to Randomized Conditions section). Participants had no way of knowing that an alternative condition existed. Therefore, participants were blind to the investigational arm they received. All data were collected using web-based surveys at 3 assessment points: baseline, 2 weeks, and 4 weeks postenrollment.

### Procedure

Participants were recruited using a mix of website, social media, and SMS text messaging methods. Specifically, a link to the study interest form was posted on the Smokefree.gov webpage for quitSTART during the entire recruitment period. In addition, a 3-week social media campaign was implemented across Smokefree.gov’s Twitter (subsequently rebranded as X), Facebook (Meta), and Instagram (Meta) accounts. In April 2021, a recruitment text blast was sent to any enrolled users of the SmokefreeTXT text messaging cessation program. Recruitment materials instructed applicants to complete a web-based interest form that included sociodemographic information and smoking history. Research staff screened for eligibility and performed identity verification of all potentially eligible participants before being contacted for informed consent. A total of 1066 interest forms were completed.

Eligible participants were required to meet the following criteria: (1) English-speaking adults who smoke cigarettes living in the United States, (2) not pregnant or trying to become pregnant in the next month, (3) owned a smartphone, (4) identity could be verified using a commercial identity verification service [48], and (5) consistent smoking information given between interest form and consent (ie, reported current smoking on both the interest and consent forms). Individuals whose identities remained unclear were contacted by study staff to determine eligibility. All eligible participants were randomized 1:1 at the time of eligibility notification using a simple randomization table in Microsoft Excel, without any stratification, then sent a notification to complete study enrollment by completing a web-based consent form. Separate consent forms were used for participants in the nonincentivized EMA arm and the incentivized EMA arm, respectively. The consent forms were identical with the exception of the compensation plan, which differed by arm (refer to Randomized Conditions section). The presence of an alternative compensation plan was not disclosed to potential participants.

After completing informed consent, all participants were sent the web-based baseline survey to complete. Following completion of the baseline survey, participants were given information about downloading quitSTART and were instructed to confirm download of the app with study staff. Participants were considered fully enrolled when they had completed the baseline survey and notified study staff that they had downloaded the app. Follow-up surveys were sent via email at 2 and 4 weeks after app download. Of 1066 completed interest forms, 572 were determined eligible and were randomized, 210 consented to participate, and 152 fully enrolled. Response rates to the 2-week and 4-week surveys were 89.5% (136/152) and 88.2% (134/152), respectively, with no differences in response rates by arm.

### Randomized Conditions

#### Nonincentivized EMA Arm

Participants randomized to the nonincentivized arm were instructed to use the app however they would like to, and that their compensation for participation depended only on survey completions at baseline, 2, and 4 weeks. Incentives in the form of electronic gift cards were dispersed after the 2- and 4-week surveys. They received all quitSTART EMA notifications but were not incentivized based on their EMA completions. They were asked to use quitSTART for 4 weeks.

#### Incentivized EMA Arm

Participants randomized to the incentivized EMA arm were informed that part of their compensation for study participation would be based on their level of EMA participation. Specifically, those with higher levels of participation received higher compensation. Participants had to complete at least half of the programmed EMAs to receive compensation for the EMA portion of their incentive and received increasing amounts for completion of 50%‐74%, 75%‐89%, or ≥90% of EMA prompts [18,29]. Participants in the incentivized EMA arm also received compensation for survey completions at baseline, 2, and 4 weeks. Incentives in the form of electronic gift cards were dispersed after the 2- and 4-week surveys. They were asked to use quitSTART for 4 weeks.

### Measures and Coding

The baseline assessment included items measuring smoking frequency (daily or nondaily smoking), use of other tobacco products (coded as “yes” for report of past 30-day use of cigar, pipe, hookah, electronic cigarette, smokeless tobacco, or snus, or “no”), nicotine dependence (measured using Fagerstrom Test for Nicotine Dependence [49], total score range: 0‐10), use of menthol cigarettes (coded as “yes” or “no”), and previous quit attempts in the past 12 months (coded as “yes” or “no”). Alcohol consumption was also assessed using number of the drinking days per week, the average number of drinks consumed when drinking, and the frequency of binge drinking. Self-efficacy to quit smoking [50], motivation to quit smoking, and indicators of anxiety and depression [51] were also collected. Demographic characteristics, including gender identity (coded as “women,” “man,” or “another gender identity” included transgender women, transgender man, nonbinary, or “something else”, combined due to small cell sizes), race (coded as “Black,” “White,” or “other”), ethnicity (coded as “Hispanic or Latino” or “not Hispanic or Latino”), education (coded as “less than high school,” “high school or GED,” “some college,” and “college graduate or more”), marital status (coded as “married or partnered,” “divorced,” “widowed,” “separated,” and “single, never married”), and sexual orientation (coded as “straight,” or “LGB+” [included gay or lesbian, bisexual, and “something else”]) was also collected.

Follow-up surveys at 2 and 4 weeks after app download assessed the primary outcome of interest: 7-day point prevalence smoking abstinence (responses to the question: “Have you smoked a cigarette [even a puff] in the past 7 days?” were “yes” and “no”). In addition, continuous abstinence (responses to the question: “In the last two weeks [four for the 4-week follow-up], have you smoked at all?” were “yes,” “no,” and “not sure,” and were coded as “yes” and “no or not sure”), number of cigarettes smoked per day (for those who continued smoking), other tobacco product use (coded as “yes” for report of past 2 week use of cigar, pipe, hookah, electronic cigarette, smokeless tobacco, or snus, or “no”), quit attempts in the last 2 weeks (coded as any, or none), any use of cessation medication, such as nicotine replacement therapy (coded as yes or no), reasons for relapse (if still smoking), motivation to quit smoking (if still smoking), anxiety and depression, and perceived usability of the quitSTART app [52] were assessed in follow-up surveys.

Participants’ total number of EMA completions was obtained from quitSTART app use data and calculated by summing the number of EMAs completed during the first 2 weeks after app download by study arm.

### Statistical Analysis

All analyses were performed using SAS 9.4. Demographic and tobacco use characteristics of the sample were summarized overall and by randomized arm. To determine the impact of incentivization on EMA participation, total EMAs completed were compared by arm using a *t* test. Given the overall study size and rarity of abstinence in the study population, Firth logistic regression modeling was used to determine the association between randomized arm and 7-day point prevalence abstinence, with 1 model for 2-week and 4-week abstinence, respectively. Given that participants were randomized before they were fully enrolled, there was potential for consent and enrollment bias. Thus, demographic and tobacco use characteristics were also compared between randomized arms using Chi-square and *t* tests as appropriate and revealed statistically significant differences in the distributions of baseline smoking frequency, use of menthol cigarettes, and use of other tobacco products between those who consented but did not fully enroll to those who fully enrolled. To account for this potential bias, we also created models adjusting for these differences. Testing for the presence of collinearity revealed that the use of menthol cigarettes and the use of other tobacco products were correlated, and therefore, only 1 variable was selected for inclusion in the adjusted model. To determine which of the 2 variables to include in the final model, we calculated the percent change in the estimate of the association between randomized arm and 7-day point prevalence abstinence to determine which potential covariate had the largest impact on the primary associations of interest (study arm and cessation). Inclusion of the use of other tobacco products resulted in a 21% and 19% change in the point estimate of the impact of arm for 2- and 4-week cessation, respectively. Inclusion of menthol cigarette use resulted in a 16% and 15% change in the point estimate of the impact of arm for 2- and 4-week cessation, respectively. Therefore, 2 adjusted Firth logistic regression models were created, including randomized arm, smoking frequency, and concurrent use of other tobacco products, for 2- and 4-week cessation. As a sensitivity analysis, unadjusted and adjusted models were also created with missing responses for cessation coded as continued smoking.

### Power

A final sample of 121 participants was selected as the target sample for this study, as it would allow for the detection of a 25% difference in smoking status between arms for cessation rates as low as 27% with an α of 0.05% and 80% power. This final sample would also allow for detection of a 2-fold difference in smoking status between arms for smoking cessation rates as low as 13%. This range of cessation rates was chosen as they fell within previously reported cessation rates in smartphone app–based cessation programs with and without EMA and recruitment from online settings [15,18,21]. We also planned for a 20% attrition rate (previous research using online recruited samples for a digital cessation intervention had attrition rates between 15% and 18%) [15,21]. Thus, an initial sample of 151 was deemed sufficient for this study.

### Ethical Considerations

The study design and protocol were approved by the Institutional Review Board at the University of Virginia (University of Virginia Institutional Review Board for the Social and Behavioral Sciences protocol 3643). All participants provided informed consent before participation and were able to withdraw their participation at any time. Privacy and confidentiality of all data were ensured at all times, including suppressing of small cell counts in reported results. As mentioned, incentives in the form of electronic gift cards were dispersed after the 2- and 4-week surveys. Total potential compensation between the 2 arms was identical (US $50).

## Results

**Figure 1.**
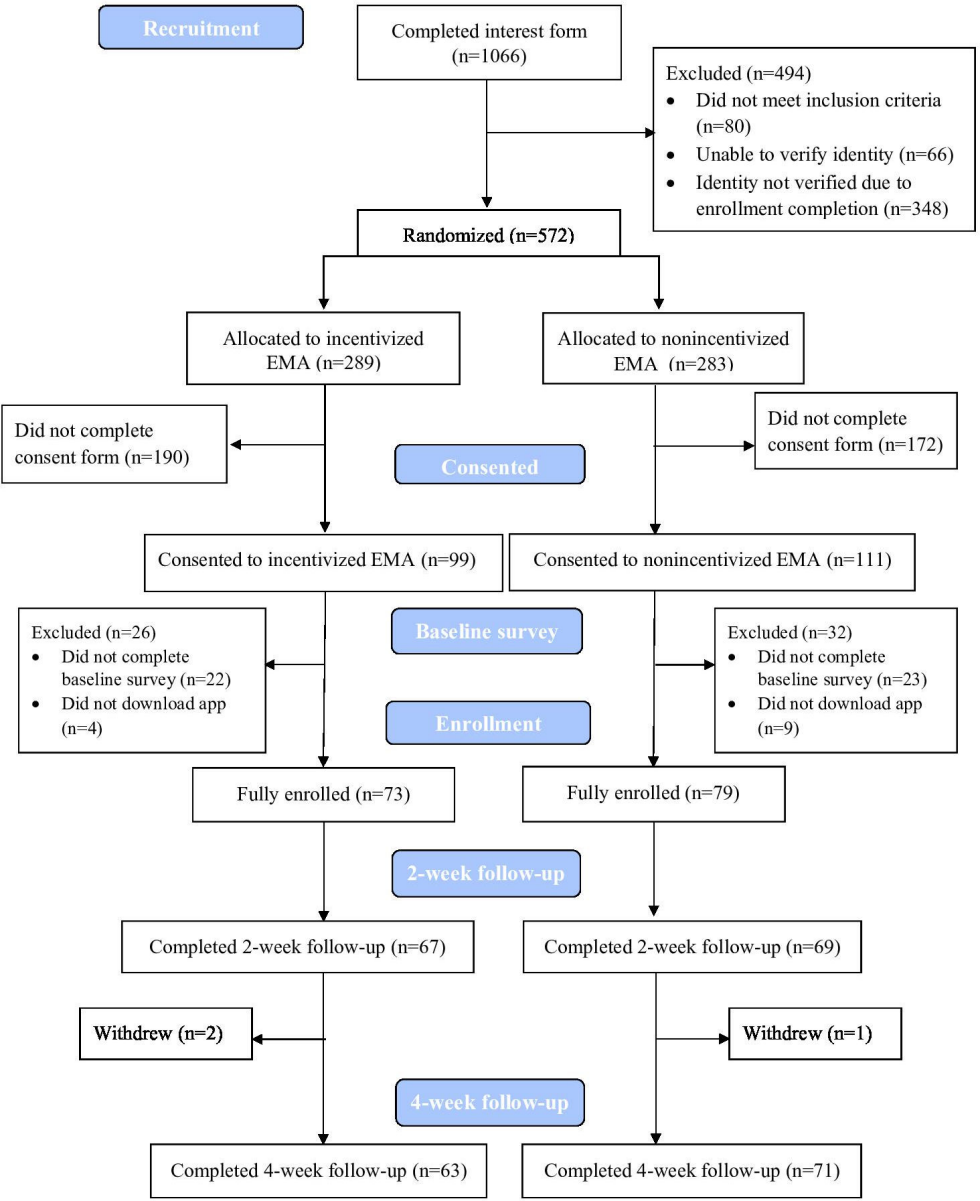
CONSORT (Consolidated Standards of Reporting Trials) diagram. The CONSORT diagram shows flow of participants through the study, from completion of an interest form to the final follow-up survey at 4 weeks. All participants who were still enrolled in the study (ie, had not withdrawn) at the time of the 4-week follow-up were offered the opportunity to participate in that survey, even if they did not respond to the 2-week survey. EMA: ecological momentary assessment.

CONSORT (Consolidated Standards of Reporting Trials) information can be found in Figure 1. Most study participants were women (116/152, 76%), White (119/152, 78%), and not Hispanic or Latino (135/152, 89%, Table 1). The average age of participants at study enrollment was approximately 45 (SD 12.45) years. Just under half (72/152, 47%) of participants had a college degree or higher education. Participants reported low-to-medium nicotine dependence with an average Fagerstrom score of 4.85 (SD 2.36). About one-third of participants reported use of other tobacco products (53/152, 35%) and use of menthol cigarettes (50/152, 33%). Less than a quarter of participants (34/152, 22%) reported having not tried to quit smoking within the last year.

**Table 1.** Demographic and baseline tobacco use characteristics overall and by study arm.

Characteristics	Total (N=152)	Incentivized EMA^a^ (n=73)	Nonincentivized EMA (n=79)
Age (y), mean (SD)	45.23 (12.45)	46.55 (11.94)	44.01 (12.86)
Gender^b^, n (%)			
Woman	116 (76)	58 (80)	58 (73)
Man	33 (22)	15 (20)	18 (23)
Race, n (%)			
Black	19 (13)	6 (8)	13 (17)
Other	14 (9)	6 (8)	8 (10)
White	119 (78)	61 (84)	58 (73)
Ethnicity, n (%)			
Hispanic or Latino	17 (11)	6 (8)	11 (14)
Not Hispanic or Latino	135 (89)	67 (92)	68 (86)
Education, n (%)			
Less than high school	6 (4)	4 (5)	2 (2)
High school or GED	15 (10)	9 (12)	6 (8)
Some college	59 (39)	26 (36)	33 (42)
College graduate or more	72 (47)	34 (47)	38 (48)
Marital status, n (%)			
Married or partnered	79 (52)	39 (53)	40 (51)
Divorced	28 (19)	14 (19)	14 (18)
Widowed	6 (4)	4 (5.5)	2 (2)
Separated	8 (5)	4 (5.5)	4 (5)
Single, never married	31 (20)	12 (17)	19 (24)
Sexual orientation, n (%)			
Straight	120 (79)	60 (82)	60 (76)
LGB+^c^	32 (21)	13 (18)	19 (24)
Smoking frequency, n (%)			
Every day	140 (92)	71 (97)	69 (87)
Some days	12 (8)	2 (3)	10 (13)
Fagerstrom total score, mean (SD)	4.85 (2.36)	4.95 (2.36)	4.77 (2.37)
Concurrent use of other tobacco products, n (%)			
Yes	53 (35)	19 (26)	34 (43)
No	99 (65)	54 (74)	45 (57)
Smokes menthol cigarettes, n (%)			
Yes	50 (33)	17 (23)	33 (42)
No	102 (67)	56 (77)	46 (58)
Quit attempt in the last 12 months, n (%)			
Yes	118 (78)	60 (82)	58 (73)
No	34 (22)	13 (18)	21 (27)

a EMA: ecological momentary assessment.

b Total does not add to 100% due to missing or suppressed values due to small sample size.

c LGB+: lesbian, gay, bisexual, and other sexual orientations.

Mean EMAs completed in the incentivized arm was 13.3 (SD 11.2, range 0‐40, average completion rate of 31.7% out of 42 total EMA prompts) and 4.7 (SD 5.8, range 0‐28, average completion rate of 11.2% out of 42 total EMA prompts) in the nonincentivized arm (*P*<.001). Cessation rates were 9% and 20.3% at 2 weeks (*P*=.06), and 17.5% and 36.6% at 4 weeks (*P*=.01) in the incentivized arm and nonincentivized arm, respectively (Table 2).

**Table 2.** Smoking cessation outcomes overall and by group.

Variable and time point	Total	Incentivized EMA^a^	Nonincentivized EMA	*P* value
2 weeks				
7-day abstinence, n (%)				.06
Yes	20 (15)	6 (9)	14 (20)	
No	116 (85)	61 (91)	55 (80)	
Continuous abstinence, n (%)				.08
Yes	17 (12.5)	5 (7)	12 (17)	
No	119 (87.5)	62 (93)	57 (83)	
4 weeks				
7-day abstinence, n (%)				.01
Yes	37 (28)	11 (17)	26 (37)	
No	97 (72)	52 (83)	45 (63)	
Continuous abstinence, n (%)				.08
Yes	18 (13)	5 (8)	13 (18)	
No	116 (87)	58 (92)	58 (82)	

a EMA: ecological momentary assessment.

At 2 weeks, incentivization was not associated with abstinence in the crude (odds ratio [OR] 0.39, 95% CI 0.14‐1.08) or adjusted models (adjusted OR 0.60, 95% CI 0.21‐1.73, Table 3). At 4 weeks, compared with participants randomized to the nonincentivized arm, those randomized to the incentivized arm had lower odds of abstinence in the crude model (OR 0.37, 95% CI 0.16‐0.82); however, this association was no longer statistically significant in the adjusted model (adjusted OR 0.51, 95% CI 0.22‐1.19). Participants who reported having used other tobacco products in the past 30 days had 3.42 (95% CI 1.27‐9.26) and 2.71 (95% CI 1.21‐6.10) times the odds of abstinence at 2 and 4 weeks, respectively. Sensitivity analyses coding missing responses as smoking found similar results.

**Table 3.** Associations between incentivization arm and cessation.

Variable and time point	Crude, OR^a^ (95% CI)	Adjusted model^b^, OR (95% CI)	Sensitivity analyses^c^, OR (95% CI)
2 weeks			
Arm			
Incentivized EMA^d^	0.39 (0.14‐1.08)	0.60 (0.21‐1.73)	0.58 (0.21‐1.64)
Nonincentivized EMA	Referent	Referent	Referent
Smoking frequency			
Everyday	Referent	Referent	Referent
Some days	4.58 (1.17‐18.03)	3.69 (0.84‐16.15)	2.97 (0.77‐11.51)
Concurrent use of other tobacco products			
Yes	3.94 (1.47‐10.52)	3.42 (1.27‐9.26)	2.78 (1.06‐7.31)
No	Referent	Referent	Referent
4 weeks			
Arm			
Incentivized EMA	0.37 (0.16‐0.82)	0.51 (0.22‐1.19)	0.47 (0.21‐1.06)
Nonincentivized EMA	Referent	Referent	Referent
Smoking frequency			
Everyday	Referent	Referent	Referent
Some days	3.56 (1.02‐12.49)	2.54 (0.67‐9.65)	2.51 (0.72‐8.81)
Concurrent use of other tobacco products			
Yes	3.21 (1.46‐7.06)	2.71 (1.21‐6.10)	2.46 (1.13‐5.38)
No	Referent	Referent	Referent

a OR: odds ratio.

b Model adjusted for all listed covariates (smoking frequency and concurrent use of other tobacco products).

c Missing respondents coded as smoking at each respective time point.

d EMA: ecological momentary assessment.

## Discussion

### Principal Findings

This study reports novel findings from a randomized trial designed to determine the isolated impact of incentivized EMA completion on smoking cessation outcomes. We found that while incentivizing EMA completion did lead to participants completing more EMAs, it did not result in increased likelihood of cessation and potentially had a negative impact on short-term cessation outcomes. Participants who reported the use of other, noncigarette tobacco products in the 30 days preceding their cessation attempt had higher odds of cessation. We hypothesized that increased EMA participation would be associated with higher rates of short-term abstinence, due to participants receiving more personally tailored content during the first 2 weeks of a quit attempt, as well as higher overall engagement with quitSTART. This hypothesis was informed by the success that previously tested apps have had in supporting cessation, where tracking is one of the most commonly included features [15,17,18,38,40,53], and a recent meta-analysis showing that increased overall engagement within cessation apps was associated with abstinence [54].

Our results are similar to McCarthy et al [55], who randomized adults who were interested in quitting smoking into 2 levels of EMA intensity (once a day vs 6 times a day). While participants in the more intensive EMA arm had decreased cravings, anger, and anxiety, there was no association between EMA intensity and 2-week cessation [55]. Given the widespread inclusion of EMA functionality in cessation apps, it is important that we understand the implications of using this feature. Future analyses should consider the impact of study arm on patterns of app use [56], as well as the association between levels of EMA completion and cessation, to more fully understand how EMA engagement influences cessation outcomes. The goal of this study was to attempt to isolate the impact of EMA engagement on cessation outcomes, without influencing participants’ initial perceptions of EMA engagement. Therefore, in an effort to avoid creating bias in participants’ perceived value of engaging in EMA by framing it in a positive or negative light, the purpose of the incentivization of EMA was not specified to participants. However, it is possible that not knowing why EMA completion was incentivized resulted in participants focusing exclusively on completing the required task rather than engaging freely with the content or other supportive features available within the app, or the process of quitting broadly. It is also possible that participants randomized to the incentivized arm experienced psychological reactance to having their use of quitSTART be prescribed [57]. Future work could consider how framing of specific features, in addition to incentivization, may influence engagement with the app itself as well as engaging in the process of behavior change.

In clinical trials, when EMA completion is necessary to inform JITAI content, participation in EMA has been necessarily incentivized [18,29]. However, once an app becomes publicly available, resources and infrastructure may not be in place to provide this type of monetary incentivization for EMA completion. Assessing the impact of EMA incentivization on cessation is of the utmost importance so that we can better understand the utility of EMA if it is not incentivized (ie, in real-world settings). This study provides evidence that incentivizing EMA completion results in greater engagement with EMAs in a real-world setting. However, the finding that greater engagement with EMAs was not associated with higher odds of cessation, and in fact potentially had a chilling effect, was unexpected. One possible explanation for this finding is that incentivizing EMA participation outside the context of a clinical trial may fundamentally alter the way that users interact with the app. It is also possible that participants in the incentivized arm had a self-perception of lower commitment to quitting if the incentivization was needed to promote app use. However, 1 metric of commitment to quitting, reported use of nicotine replacement therapy or other cessation medication aid, was similar between arms at each follow-up assessment, indicating potentially similar investment in quitting during this study. In addition, 99% (150/151) of participants reported intending to quit smoking in the next 30 days at baseline (data not shown). Nevertheless, the impact of incentivizing EMA completion on cessation outcomes could vary depending on what it is adding qualitatively to an individual’s cessation process (eg, mood support vs tracking cravings). It is also important to consider the potential influence of incentivization on what users are reporting during EMAs, as EMAs being used to support JITAIs depend on a low prevalence of reporting bias in order to be effective. Future mixed methods research could also help by providing new insights into users’ perceived utility of EMA engagement, including what constructs users believe would be useful or important to monitor and identify alternative or stronger incentives to encourage engagement.

People likely need to use a combination of features to help them quit, and which features best support a given person in their quit attempt could differ [56]. Therefore, more research is also needed on the extent to which incentivizing a range of app features impacts cessation and engagement with other app features, and if there are specific subgroups who would particularly benefit from incentivization. In addition, quantifying the impact of complex patterns of feature use (incentivized or not) with cessation could identify new target features for incentivization. Overall, these results provide preliminary evidence that within a publicly available app, allowing autonomy with the use of app features versus directed app use may result in better outcomes.

While this study provides important real-world data on EMA participation, the results cannot be generalized to all cessation apps using EMA. Furthermore, our results should not be interpreted as an indication that EMA is not a useful feature or that high engagement is still not a worthwhile goal. This is because interventions using this feature may vary in the purpose of including EMA, and therefore, how an EMA protocol is implemented and the importance of compliance. For example, in a study by Businelle et al [18], the EMA component lasted for 5 weeks, and data collected from the EMA were used in a JITAI to prevent relapse. In a study by Bricker et al [38], reporting repeated cessation success in a daily EMA unlocked additional intervention content. EMA protocols also differ in what they measure (eg, cravings versus mood). In addition, there are differences in whether or how information entered during EMA completion is presented back to participants for tracking, self-monitoring, or reflection. In quitSTART, users can track money saved by not smoking, number of EMAs completed, reports of cravings, and slips and mood reported outside of the EMAs; they are unable to view the information previously collected during each EMA, which may inherently limit the impact of EMA completion on tracking and self-monitoring. However, quitSTART users who complete more EMAs receive more frequent tailored messages to their responses at the time of each completion. Future research is needed to understand the optimal way in which to implement EMA functionality in research and real-world settings, which will likely depend on the expected impact of EMA completion on cessation.

Concurrent use of other tobacco products was associated with higher odds of short-term cessation outcomes in this study. However, we do not have detailed information on how long participants had been using these products or their motivation for using them. Among participants who used other tobacco products at enrollment, 81% (43/53) used only 1 additional product, and the most commonly reported other products used were electronic cigarettes and cigars. Use of vaped nicotine products has been reported by adults who currently smoke as a cessation aid used to quit smoking in previous research [8], but the motivation for poly tobacco product use among participants in this study is unknown. Previous literature has indicated that poly tobacco product use is associated with lower long-term smoking cessation success [58], making it important to continue to measure and determine the impact of poly tobacco use on short- and long-term cessation outcomes.

### Limitations

This study has several limitations that need to be considered. First, we were powered to detect a difference in smoking abstinence of at least 25% between groups for cessation rates at 27% or higher. While we successfully recruited the sample needed to reach this goal, and response rates were high, cessation rates were lower than estimated. Therefore, our power may have been reduced to determine the impact of incentivization on short-term cessation. Second, this study only examined short-term (2- and 4-week) cessation; however, early cessation success is predictive of longer-term success [21,59-61]. Therefore, our results pertaining to the impact of EMA completion on early cessation are still relevant to those who are interested in developing successful cessation apps. This study considered EMA engagement as a primary outcome of interest, but we were not able to evaluate the accuracy of data provided by participants when they were responding to an EMA prompt. However, participants were not instructed on how to answer EMAs, and their responses did not impact the incentive amount they received. This may mitigate reporting bias. Finally, our results are self-reported and could have been affected by participant misreporting. However, we did employ sensitivity analyses to capture the potential impact of continued smoking among participants who did not respond to the 2- or 4-week follow-up surveys. Our study also includes notable strengths. Particularly, we recruited from the Smokfree.gov Initiative web and mobile platforms; thus, our sample draws from real people who smoke and are interested in using digital tools for cessation support [42]. This study contributes to the growing literature around smartphone app–supported cessation using a widely used, publicly available program. This study highlights how user data derived from interactions with real-world cessation apps can inform future research on the potential population health impact of these programs, as well as inform stakeholders who are engaged in program planning, design, and implementation.

### Conclusions

This study attempted to isolate and examine the effect of incentivizing EMA engagement on smoking cessation success for adults who smoke using a smartphone app to quit. Our findings suggest that there is no additional short-term cessation benefit conferred by incentivizing EMA completions. Our results are focused on our primary outcome of interest, smoking cessation, but more research is now needed to better understand if EMA incentivization could have influenced responses to the EMA itself or use of other features within quitSTART. Future studies are also needed to continue to identify smartphone app features that are the most supportive of quit attempts and the efficacy of EMA participation for cessation among specific user groups.

## Supplementary material

10.2196/67630 Multimedia Appendix 1 Tailored content based on ecological momentary assessment responses.

10.2196/67630 Checklist 1 CONSORT-eHEALTH V1.6. CONSORT: Consolidated Standards of Reporting Trials.

## Abbreviations

CONSORT: Consolidated Standards of Reporting Trials
EMA: ecological momentary assessment
JITAI: just-in-time adaptive intervention
mHealth: mobile health
OR: odds ratio
