# Supplementary material for: Impact of Ecological Momentary Assessment Participation on Short-Term Smoking Cessation: quitSTART Ecological Momentary Assessment Incentivization Randomized Trial
Source: J Med Internet Res. 2025 Jul 18;27:e67630. doi: 10.2196/67630 (PMC12294643; doi:10.2196/67630)
Supplement: Multimedia Appendix 1 [file jmir-v27-e67630-s001.docx]

| **Supplemental Table 1. Tailored content based on EMA responses.** | | |
| --- | --- | --- |
| **If response is…** | **Then…** | **Example Tailored Content** |
| “Happy” or “Relaxed” and Craving < 5 | Happy content | Spread joy: Feeling good today? Spread happiness with those around you. One small kindness to another person can change the whole day. |
| “Angry”, “Stressed”, or “Sad” and Craving < 5 | Mood content | Stress is…stressful, but your quit is too. It’s important not to let it wear you down. Take control! Flex your strength but take care of you – get a glass of water, exercise. |
| “Happy” or “Relaxed” and Craving >= 5 | Craving content | Starve your craving: Don’t feed a craving by smoking! Starve it. Every time you resist the urge to smoke, your craving gets weaker, so stick to your smokefree plan. |
| “Angry”, “Stressed”, or “Sad” and Craving >= 5 | Random selection of mood or craving content | Tailored mood or craving content as described above. |
